# The relationship of the shear wave elastography findings of patients with unilateral lumbar disc herniation and clinical characteristics

**DOI:** 10.1186/s12891-019-2814-7

**Published:** 2019-09-29

**Authors:** Qingyuan Wang, Hao Zhang, Jinxin Zhang, Hanqi Zhang, Hui Zheng

**Affiliations:** 1 Department of Ultrasound, the First Affiliated Hospital of Anhui Medical University, No. 218 Jixi Road, Shushan District, Hefei, Anhui China; 2 Department of Neurosurgery, Heidelberg University Hospital, Im Neuenheimer Feld, 400 Heidelberg, Germany

**Keywords:** Ultrasound, Sciatic nerve, Shear wave elastography, Lumbar disc herniation

## Abstract

**Background:**

The aim of this study was to find out whether the shear wave elastography (SWE) findings of patients with unilateral lumbar disc herniation (LDH) were related to clinical characteristics.

**Methods:**

For this purpose, the study group included patients (*N* = 20; 13 male, 7 female) with complaints of unilateral sciatica, with foraminal stenosis caused by one level of LDH (L4-L5 or L5-S1). An gender-and age-matched control group (*N* = 27; 16 male, 11 female) was included. All the patients were examined on both the axial and longitudinal planes bilaterally at the same level using a convex array probe (1- 6 MHz, Supersonic Imagine, Aix en Provence, France).

**Results:**

The sciatic nerve stiffness measured on longitudinal planes of the affected side was significantly higher than unaffected side (*p* < 0.001) and the control group (*P* < 0.05). Furthermore, the symptom duration of unilateral LDH is positively correlated with the stiffness the sciatic nerve (r = 0.52, *p* = 0.019).

**Conclusion:**

According to these findings, ultrasound imaging can be considered as a useful tool to detect changes in the sciatic nerve due to disc herniation. This technique will have a promising prospect for many patients with unilateral LDH in monitoring stiffness during rehabilitation and before or after surgery.

## Background

Many studies have shown that about 80% of the population suffers from low back pain (LBP) [1]. There are a variety of reasons for LBP, the most common reason is degenerative disc disease and LDH due to intervertebral degeneration [2]. Compression of the lumbosacral nerve roots by a herniated disc contribute to LBP. Nearly 40% of these patients sustain radicular pain along the sciatic nerve distribution [3]. These spine-related expenditure have steeped increased recently in the USA [4]. Because its prevalence and significant impact on personal life and social life, early diagnosis of LDH and assessment of sciatic nerve compression are of substantial significance.

Ultrasound is the most widely used imaging technique for clinical work due to its convenience, high repeatability and lack of ionizing radiation. Recently, sonoelastography, a noninvasive method for quantification of the stiffness of tissue, has also been reported as a promising technique for the detection of changes in the peripheral neuropathy. There are two primary techniques:strain elastography (SE) and SWE [5]. SE visualizes tissue deformation with compression applied by the examiner, and in SWE, shear waves are produced by the transducer [6]. SWE which calculates Young’s elastic modulus may give accurate values of stiffness in selected areas inside the box of the measurement, giving us the result in kilopascal (kPa) [7, 8]. Due to its particular mechanical relationship with lumbar disc, it was thought that as the history of LDH increases, the stiffness of the sciatic nerve will change. Therefore, the aim of this study was to examine the elastographic findings of the sciatic nerve in patients with unilateral LDH.

## Methods

The study group included twenty patients with complaints of unilateral sciatica, with foraminal stenosis caused by one level of LDH (L4-L5 or L5-S1). A gender-and age-matched control group (*N* = 27; 16 male, 11 female) was formed healthy participants. Healthy participants did not have any indication of the sciatic nerve involvement. Participants who met any of the following criteria were excluded:diabetes mellitus, any type of polyneuropathy, trauma, previous history of back surgery, or piriformis syndrome. Participants with conditions that reduce image quality on US imaging (thick gluteal subcutaneous fat tissue, hip joint prosthesis and/or degeneration) or professional athletes were also excluded. Every subject were selected from those with MRI results in the department of spine surgery. All the patients underwent physical examination (manual muscle testing, sensory testing, and spine SLR test), a visual analog scale (VAS). Each subject was informed of the methods and procedures of this study. Every patient signed a consent form. The study protocol was approved by the local ethics committee.

**Fig. 1:**
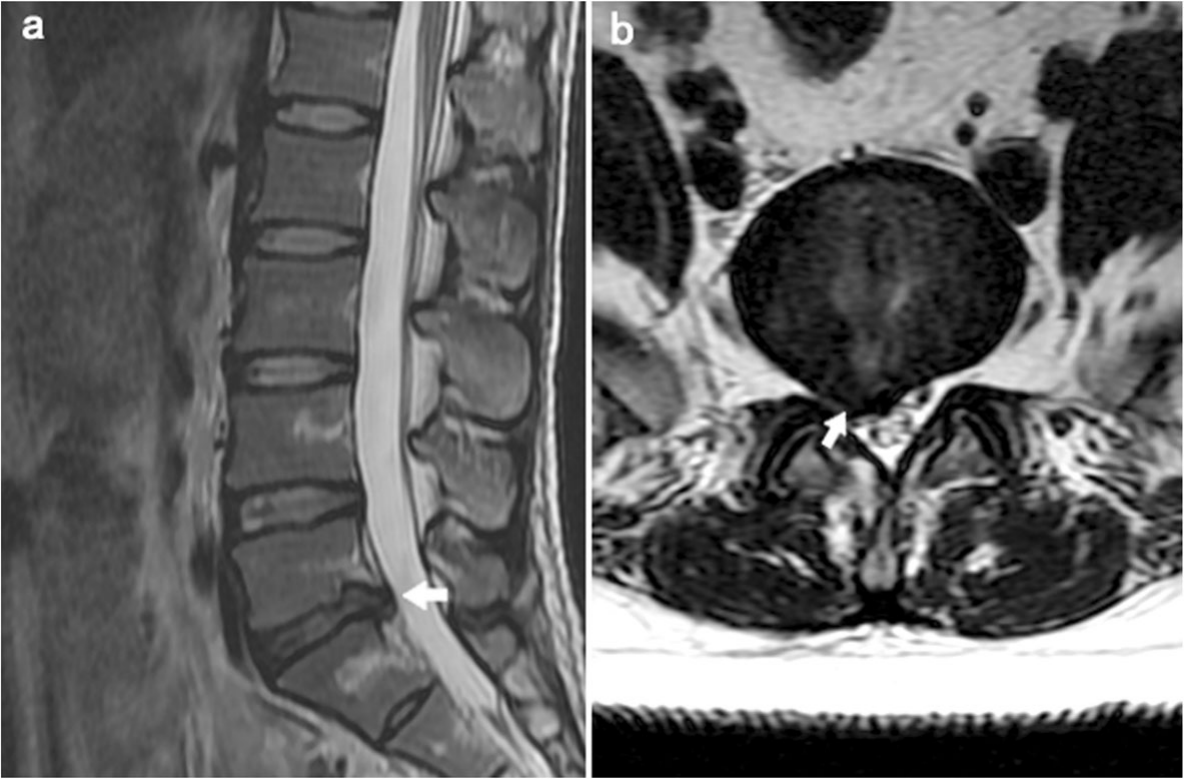
A 25-year-old male patient with complaints of sciatica for 10 months (**a**: T2WI sagittal, **b**: T2WI axial view), MRI illustrates L5/S1 disc herniation and right nerve root compression (white arrow)

Patients demographic data (gender、age、BMI、symptom duration) were recorded. MRI results including level of herniation、herniation type、classification of foraminal stenosis and nerve-root pressure (Fig. 1).

**Fig. 2:**
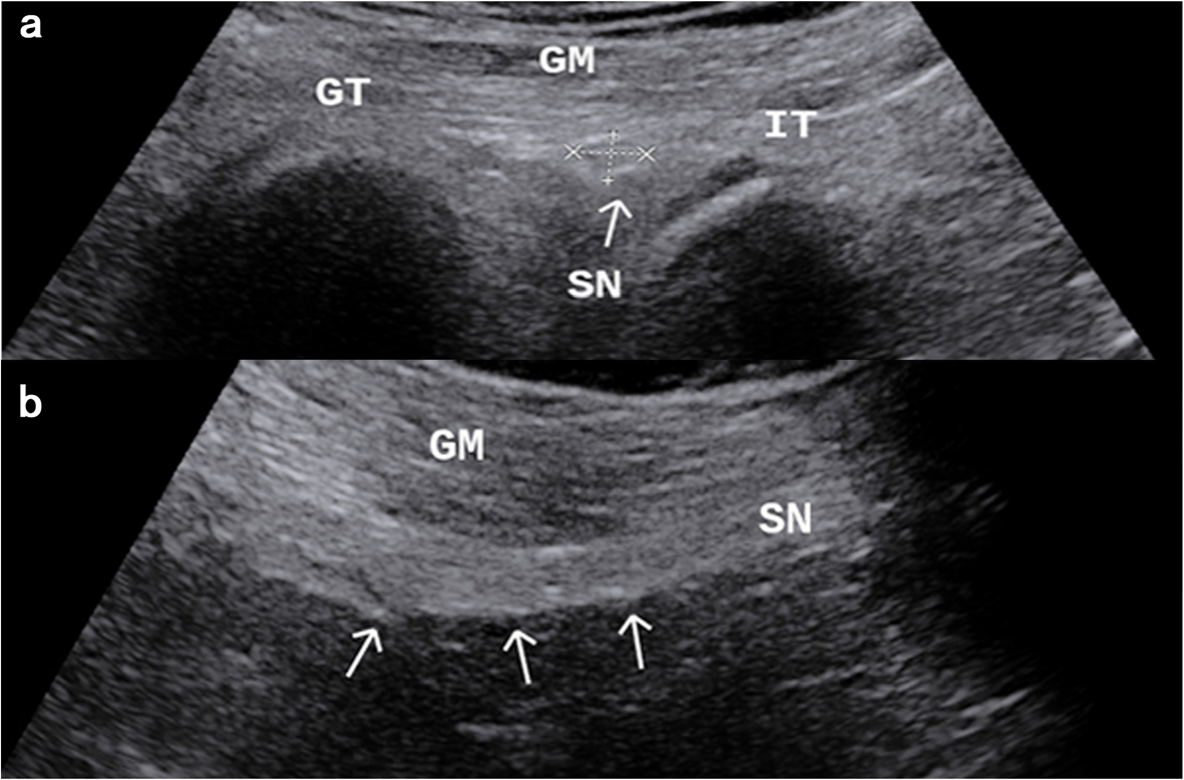
Subgluteal approach sonographic appearance in axial plane (**a**) and subgluteal approach sonographic appearance in longitudinal plane (**b**). (GT: Great trochanter, IT: Ischial tuberosity, GM: Gluteus maximus, SN: Sciatic nerve (arrows))

All the patients were evaluated by an experienced sonographer, with more than one thousand patients of experience. The elastographic examination was performed with a Supersonic Aixplorer Ultrasound system (Supersonic Imagine, Aix en Provence, France) equipped with elastographic software and a 1-6 MHz convex array transducer. Coupling gel was used between the transducer and the skin to obtain images. Every subject was examined on both the axial and longitudinal planes bilaterally from subgluteal region to popliteal fossa in prone position during plantar flexion for the knee 180° condition. The sciatic nerve was identified on the axial plane of the subgluteal region and the sciatic nerve diameters (thickness and width) was measured. Then, the transducer was rotated 90° to obtain a longitudinal imaging plane at the same level (Fig. 2). Once the sciatic nerve was visualized on the longitudinal plane of the subgluteal region, the shear wave elastography examination was performed at the same level without compression. The operator kept the transducer stationary during SWE imaging acquisitions. The B-mode and elastographic images were shown simultaneously side by side on a split-screen monitor. The quantitative analysis of sciatic nerve stiffness with SWE used kilopascal measurement with a color scale of 0 to 70 kPa and a circular region of interest at an interval of 4 mm. The shear wave elastic modulus for each sciatic nerve was obtained in at least 3 measurements, and the average values were used for the statistical analysis. The images of each subjects were examined by the same experienced sonographer, the images were analyzed by another sonographer, and the two sonographers were not aware of the clinical case data of the subjects.

The IBM SPSS software (version 16.0; IBM Corporation, New York, USA) was used for the statistics analyses. All data are reported as mean ± standard deviation (SD). The normal distribution of the variables was examined using visual (histogram and probability plots) and analytical methods (Kolmogorov–Smirnov tests). Between-group comparisons were made using the Student’s t Test. The statistical significance was set at *P* < 0.05.

## Result

Demographic and clinical characteristics of each subject were shown on the Table 1. Mean symptom duration ± SD was 66.6 ± 32.0 (range, 0.25–90) months. Mean VAS scores ± SD was 6.6 ± 2.1 (range, 1–9). No significant difference was observed between the groups in terms of age (*P* = 0.779)、BMI (*P* = 0.388)、and diameter of the sciatic nerve (all *P* > 0.05).

**Table 1:** demographic and clinical characteristics

Variables	Study group (*N* = 27)	Control group (*N* = 20)	*P*
Age (years)	44.5 ± 12.9	42.1 ± 9.7	0.779
Gender			
male	7 (35)	11 (40.7)	
female	13 (65)	16 (59.3)	
BMI	24.0 ± 3.2	23.1 ± 2.6	0.388
Duration (months)	66.6 ± 32.0	–	
VAS scores	4.9 ± 2.4	–	
affected side			
Right	8 (40)	–	
Left	12 (60)	–	
Left			
Width (mm)	8.5 ± 1.2	8.8 ± 1.6	0.370
Thickness (mm)	7.2 ± 1.5	7.2 ± 1.7	0.703
Right			
Width (mm)	9.1 ± 1.7	8.4 ± 1.3	0.151
Thickness (mm)	7.2 ± 1.3	7.1 ± 1.6	0.555

The data are shown as mean ± standard deviation or *n*, (%)

**Fig. 3:**
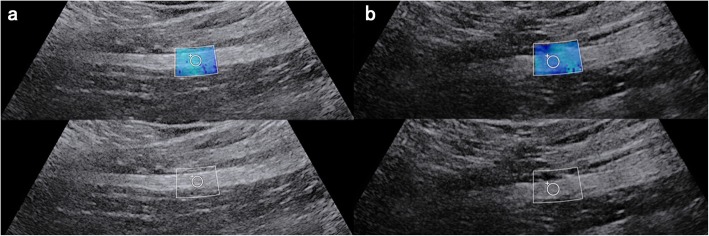
A 30-year-old male patient with complaints of sciatalgia for 12 months and VAS scores was 6. The shear wave elasticity value is measured as 22.3 kPa in the affected side (**a**), and 15.1 kPa in the unaffected side (**b**)

**Fig. 4:**
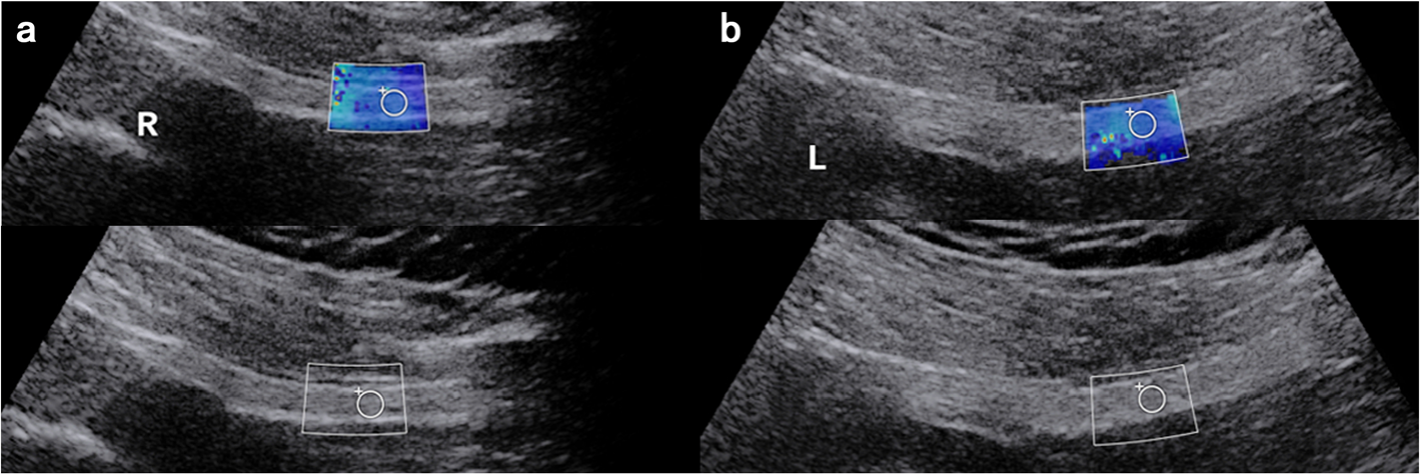
A 47-year-old female healthy subjects. Shear wave elasticity value is measured as 13.7 kPa in the right (**a**) and 13.1 kPa in the left (**b**)

US measurements were summarized on the Table 2. The affected side comprised 20 patients with mean value of 20.4 ± 4.6 kPa, and the mean value of unaffected side was 12.9 ± 2.2 kPa (Fig. 3). The control group involved 27 subjects with mean value of 12.7 ± 2.1 kPa (left) and 13.5 ± 1.9 kPa (right) (Fig. 4). The sciatic nerve stiffness measurement was found to be significantly higher on the affected side than the unaffected side (*P* < 0.05) and the control group (all *P* < 0.05). However, the difference was not statistically significantly in respect of the sciatic nerve diameter on the affected side between the unaffected side and the control group.

**Table 2:** Diameter of the sciatic nerve and shear wave elastography findings of the groups

Study group (*N* = 20)	Mean ± SD	Max-min	*P* value
Elasticity (kPa)			
affected side	20.4 ± 4.6	27.5–11.4	*P* < 0.05*
unaffected side	12.9 ± 2.2	17.3–8.9	
Width (mm)			
affected side	8.7 ± 1.6	11.3–6.5	*P* = 0.190
unaffected side	8.0 ± 1.6	11.3–5.6	
Thickness (mm)			
affected side	7.2 ± 1.2	9.9–5.4	*P* = 0.305
unaffected side	6.7 ± 1.5	9.3–4.2	
Control group (*N* = 27)			
Elasticity (kPa)			
left	12.7 ± 2.1	17.7–8.0	*P* = 0.180
right	13.5 ± 1.9	17.6–9.6

Moreover, the stiffness measurements of the affected side were found to correlate to symptom duration (r = 0.60, *p* = 0.006). Although the symptom duration and VAS scores were correlated (r = 0.52, *p* = 0.019), the sciatic nerve diameters were not correlated either with VAS scores or symptom duration (*P* > 0.05). The r value means correlation coefficient which is a non-dimensional index for measuring the linearity between two variables; the *p* value is the test value, which is to test whether the two variables have the same correlation with the sample in the population from which the sample is derived Table 3.

**Table 3:** Relationship between SWE measurements data and clinical features

	VAS scores		Duration	
	*r*	*p*	*r*	*p*
Elasticity	–	> 0.05	0.60	0.006
Diameter	–	> 0.05	–	> 0.05

## Discussion

There have been some studies in literature evaluating the sciatic nerve with elastography. In 2013, Santos claimed that sciatic nerve can be visualized and evaluated by US elastography [9]. After that, Andrade assessed the elasticity of sciatic nerve during passive movements, using SWE. It was demonstrated that the sciatic nerve stiffness can be assessed accurately using elastography, and the stiffness of the sciatic nerve can be affected by the lower limb movements [10].

This study was performed to observe the relationship of the SWE findings and clinical characteristics in patients with unilateral LDH. To the best of our knowledge, this is the first time that the correlation of the stiffness measurements and symptom duration was explored. Our study demonstrated that the longer the unilateral LDH duration, the higher the stiffness of the sciatic nerve. In addition, we also investigated the relationship of VAS scores and symptom duration. We observed that longer history of the unilateral LDH are significantly associated with more serious pain. These findings showed that the importance of early detection of LDH.

LDH, which is manifested by low back pain or sciatica, is one of the common causes of disability in the community [11]. In LDH, nucleus pulposus (NP) material exits the disk space area through the annulus fibrosus (AF), causing mild compression on the spinal nerves and resulting in neurological dysfunction including pain, sensory deficits, and weakness in the low back and leg [12]. There are several changes in the biology of the intervertebral disc which is thought to contribute to LDH. These include increased percent of type I collagen within the NP and inner AF [13], reduced water retention in the NP [14–16], degradation of collagen and extracellular matrix materials (ECM) [17], and upregulation of systems of degradation such as apoptosis, matrix metalloproteinase (MMP) expression, and inflammatory pathways [18]. Increased expression of chemokines are associated to clinical severity of sciatic pain in lumbar disk herniation patients [19] .Mechanical compression and accompanying chemical stimulation may cause severe nerve root damage, which in turn affects the axonal transport, affecting the circulation of nerve roots and the metabolism of neurotransmitters [20]. Thereby causes neurilemma edema, fibroblast infiltration, and nerve fiber deformation. These changes can lead to demyelination of the nerve and can alter the nerve connective tissue [21], causing the proliferation of scar tissue and the acceleration of the transverse wave in the nerve. SWE is an imaging technique that emits transverse waves to tissue through a transducer. According to E = 3ρc∧2 (E is the Young’s modulus, c is the shear wave propagation velocity, and ρ is the tissue density), the machine displays the E value, The faster the shear wave propagation speed, the higher the E value. SWE can quantitatively calculate the E value according to the shear wave propagation velocity in the region of interest, and evaluate the elastic properties of tissues qualitatively by the E value. The tissue stiffness is displayed by color coding. The harder the tissue, the faster the shear wave travels, the higher the E value, and the image appears red. Conversely, the smaller the E value, the softer the tissue and the blue color of the image. In this study, the sciatic nerve of affected appeared blue-green, while the normal one appeared blue. In addition, axonal injury and demyelination occur in parallel with the severity of the injury. Increased expression of chemokines is closely related to the clinical severity of sciatica in patients with LDH [19].

### Limitations

There are a couple of tissues that have to be mentioned and discussed in this study. First, the sample size is small, and the method is not suitable for thick gluteal subcutaneous fat tissue. Second, demographic data such as body mass index、walking habit should be considered. Third, a study, which evaluated the cross-sectional area of the sciatic nerves with tracing method, observed the sciatic nerve enlargement in patients with unilateral sciatica [3]. In contrast, this study showed no significant difference between the affected and unaffected sides in respect of the sciatic nerve diameter. The difference in this measurement method can be attributed ed. as a limitation. The tracing method is more sensitive and accurate than other methods to measure nerve size. Fourth, Andrade demonstrated that the stiffness of the sciatic nerve can be affected by the lower limb movements [10]. This study observed the sciatic nerve in prone position during plantar flexion for the knee 180° condition. Therefore, other position and lower limb movements should be considered.

## Conclusion

In summary, we showed that the stiffness measurements of the sciatic nerve were significantly higher in patients with LDH than in healthy subjects. In addition, this study demonstrates that the shear wave elastography of the sciatic nerve in patients with LDH is influenced by symptom duration. Moreover, VAS scores is positively correlated with symptom duration. Although this preliminary study show that shear wave elastography can detect the relationship of the sciatic nerve stiffness、symptom duration and VAS scores, further investigations are required to determine the clinical utility of this technique.

## Data Availability

The datasets used and/or analyzed during the current study are stored in our hospital and are available from the corresponding author on reasonable resquest.

## Abbreviations

AF: Annulus fibrosus
BMI: Body mass index
ECM: Extracellular matrix materials
LBP: Low back pain
LDH: Lumbar disc herniation
MMP: Matrix metalloproteinase
MRI: Magnetic resonance imaging
NP: Nucleus pulposus
SE: Strain Elastography
SLR: Straight Leg Raise
SWE: Shear wave elastography
VAS: Visual analog scale
